# A single-center, nonblinded, clinical trial comparing blood pressures before and after tourniquet application in healthy humans: A study protocol

**DOI:** 10.1371/journal.pone.0280139

**Published:** 2023-01-06

**Authors:** Samuel W. Seigler, Kristen M. Quinn, Heather L. Holman, Joshua Y. Kim, Taufiek K. Rajab

**Affiliations:** 1 College of Medicine, Medical University of South Carolina, Charleston, South Carolina, United States of America; 2 Department of Surgery, Medical University of South Carolina, Charleston, South Carolina, United States of America; 3 Human-Centered Design, Medical University of South Carolina, Charleston, South Carolina, United States of America; 4 Department of Pediatric Cardiothoracic Surgery, Medical University of South Carolina, Charleston, South Carolina, United States of America; Kurume University School of Medicine, JAPAN

## Abstract

**Introduction:**

Cardiac arrest is the leading cause of natural death in the United States, and most surviving patients suffer from neurological dysfunction. Although this is recognized as a problem, there have been very few changes to the cardiopulmonary resuscitation (CPR) procedure. Tourniquets have been recognized for their ability to increase truncal blood pressure and have been shown to improve CPR outcomes in animal models. However, the relationship between tourniquet application and blood pressure elevation has not been adequately explored in healthy human adults.

**Objectives:**

The objective of this study is to demonstrate that bilateral, non-invasive, peripheral vascular occlusion in the thighs results in an increased proximal systolic blood pressure ≥ 10 mmHg.

**Methods:**

This is a single-center, non-blinded clinical trial. Volunteers will be screened for eligibility at least 24 hours before the day of the trial. On the day of the trial, volunteers will undergo an informed consent process. If they choose to participate in the trial after informed consent, their baseline blood pressure will be measured. Volunteers will then have a Combat Application Tourniquet (CAT) applied to each thigh, and the windlasses will be tightened by IRB-approved personnel. Once no pulse can be felt in the lower extremity, blood pressure will be measured in the arm. This will be replicated three times, and the tourniquets will be loosened between trials to allow the volunteers to rest. Any complications that arise during the trial will be handled by the physician that is present.

**Analysis:**

Changes in systolic blood pressure and diastolic blood pressure will be analyzed using a Shapiro-Wilk test. Then, a one-way repeated measures analysis of variance (ANOVA) will be performed with a Holm-Sidak post-hoc test to determine the mean differences. The significance level will be set to 5% for statistical significance.

**Registry and registration number:**

Clinicaltrials.gov, NCT05324306.

## Introduction

### Background

For decades, the fields of trauma, orthopedics, and cardiology have utilized vascular occlusion devices for either hemorrhage control or to increase central pressure [1–3]. Due to their success in maintaining blood flow proximally during shock scenarios, vascular occlusion devices have increasingly been studied during non-traumatic cardiac arrests. There have been eight preclinical studies in animals that have demonstrated endovascular balloon occlusion devices significantly increase coronary artery blood flow and pressure during cardiac arrest [4–11]. Tourniquets are a non-invasive alternative to endovascular balloon occlusion devices, and they are often used to achieve vascular occlusion in clinical practice for hemorrhage control during extremity surgery and trauma. They have been well studied in civilian and military research for their safety and efficacy, but their use as a CPR adjunct has yet to be realized [12–14].

The fluid dynamics Hagen-Poiseuille Equation demonstrates in physical law why peripheral vasoconstriction increases the flow in conserved vascular networks. The Hagen-Poiseuille Equation (***Q*** = ΔPπ*r*4) gives the volumetric flow rate (*Q*) in a cylindrical tube as it relates to the viscosity of the fluid (*v*), length of the system (*L*), radius of the tube (*r*), and pressure gradient across the tubing (Δ*P*). Tourniquets placed on the legs would decrease the length of the system (*L*), thereby increasing the flow (*Q*) as these variables are inversely proportional. While this equation is an obvious oversimplification, we find it models our expectant results and confirms what we intuitively and anecdotally have noted in our patients. Beyond theory, studies in large animal models and clinical trials offer additional proof of concept [4, 12, 13, 15–19]. Pigs receiving CPR with a tourniquet adjunct experienced significantly increased blood flow to vital organs (coronary artery and carotid artery), systolic and diastolic blood pressure, end-tidal carbon dioxide, and survival rate than controls [17]. Increased systolic and diastolic blood pressure during CPR is particularly important, as these measurements have been shown to correlate with clinical outcomes. In an animal model of cardiac arrest, higher diastolic blood pressure during CPR were associated with a higher survival rate [20]. Increased rates of end organ damage and death have also been observed in humans when mean arterial pressure decreases to less than 40 mmHg, associated with a systolic blood pressure of around 55 mmHg [21].

### Rationale

To our knowledge, blood flow or pressure to vascular beds supplying vital organs (i.e., brain, heart) has not been measured in healthy adult subjects following administration of a non-invasive peripheral vascular occlusion device. This proof-of-concept data is necessary for future development and hypothesis testing for novel CPR adjuncts, which could improve survival and neurologic recovery following cardiac arrest.

### Choice of comparators

Our goal is to acquire proof of concept data demonstrating increased blood pressure in the upper extremities as a read-out for perfusion of the heart and brain following tourniquet administration on the lower extremities. This data will be critical in the development of novel CPR adjuncts. To complete our hypothesis testing safely, we plan to deploy well-studied and FDA-approved combat application tourniquets (CAT) on volunteer healthy adult subjects while measuring blood pressure and heart rate in the upper extremities. CAT tourniquets were developed in 2005 by Mark Esposito et al. (US Patent numbers 7,842,067 and 7,892,253) and soon after obtained FDA approval. CAT tourniquets have been indicated for use in emergency situations revolving around a wounded limb, such as hemorrhage control [22, 23]. CAT tourniquets are the official tourniquet of the US Army since 2005. Research participants in this study will have their blood pressure and heart rate measured in their upper extremities before and after tourniquet administration to serve as within-subject controls.

### Objectives

We intend to demonstrate that non-invasive, peripheral vascular occlusion increases blood pressure in the conserved vasculature in healthy humans while maintaining a baseline heart rate. Data from this study will provide proof of concept for the development of a novel device to improve cardiopulmonary resuscitation (CPR) outcomes during non-traumatic cardiac arrest. We hypothesize that peripheral vascular occlusion of non-vital vascular beds (the lower extremities) will increase blood flow to the more vital organs, the heart and the brain, leading to an improvement in survival and neurologic recovery after cardiac arrest as these outcomes are dependent on heart and brain ischemic injury. By using commercially available, FDA-approved tourniquets on healthy humans to measure blood pressure and heart rate with peripheral vascular occlusion, we will be testing our hypothesis in the safest way.

## Materials and methods

### Study setting

Study participants will meet in a specified conference room at the Medical University of South Carolina where the research protocol will be completed.

### Eligibility criteria

#### Inclusion criteria

1. Healthy adults with no known chronic medical conditions between the ages of 18 and 60 years old willing and able to consent themselves.
2. Subjects who are native English speakers or self-report as fluent in English.

#### Exclusion criteria

1. Subjects who have a history of or are a current smoker, have a history of or current illicit drug use, have a history of or current alcohol abuse.
2. Subjects who self-report as pregnant.
3. Subjects who self-report diabetes, cardiac disease (including hypertension) requiring medication, or a history of compartment syndrome or peripheral vascular disease.
4. Subjects with known chronic diseases not listed above, including but not limited to Rheumatoid Arthritis, McArdle’s disease, etc.
5. Subjects with elevated blood pressure (>130/80mmHg) on the day of the study.

### Interventions

#### Intervention description

Each participant’s height and weight will be recorded, and they will have their blood pressure and heart rate measured in each arm by an automated sphygmomanometer while lying down on a medical bench (baseline must not be >130/80 mmHg). This will allow each research participant to act as their own control for the experimental procedure. The measurements will be recorded by IRB-approved personnel. Then, one CAT will be placed around each thigh and tightened until no pulse can be felt in the lower extremity. Blood pressure and heart rate measurements will be repeated in each arm once the tourniquets are secured. Once these measurements are documented, the tourniquets will be removed. The maximum time the tourniquets will be applied is 5 minutes to ensure ample time for blood pressure recording. If the measurement cannot be taken within the time frame, the tourniquets will be released. The participants will rest 3–5 minutes between each repetition of the tourniquet application. The whole process will be repeated three times for data validity. CATs have been extensively studied for safety, and no long-term consequences or complications have been noted for up to two hours of continuous use.

The research team will prioritize the safety and comfort of the participant and will limit the tourniquet application time to a maximum of 5 minutes. Participant data and personal information will be kept in a secure database with a password-protected server. The data will be coded and a linking document containing any identifiers will be kept separate from the research data. Only IRB-approved personnel will have access to the final trial dataset. Upon completion, the deidentified data will be available in a separate manuscript.

After the data is collected, a debrief session will be conducted, allowing for additional subject monitoring after removal of the tourniquets. During the debrief session, the participant will be asked to lay down or sit in a relaxed position for 5 minutes or until they feel that their leg function is back to baseline. If there is any concern from the participant, the PI or IRB-approved personnel who are medical doctors, will conduct an evaluation. If any of the safety endpoints are noted, it will be recorded, and the participant will be taken to the emergency room for full examination and work-up. The PI or IRB-approved personnel will follow-up with the participant remotely until the safety concern is resolved.

#### Modifications

Research subjects may refuse to participate or stop participating in the study at any time by notifying the principal investigator or approved IRB personnel. If research participants would like to have their data removed from the study, they must notify the principal investigator in writing within a week of completing the study. If the principal investigator or approved IRB personnel determine that participation in the study is not in the best interest of the research subject, they may remove the research participant from the study.

### Outcomes

A cardiac arrest porcine model that utilized an intra-aortic balloon catheter for occlusion of the aorta at the level of the diaphragm demonstrated a mean increase in systolic pressure of 17mmHg [18]. Extrapolating from this data to our more distal point of occlusion at the lower extremities, we define the primary study endpoint as the successful application of CAT tourniquets in normal physiology in healthy humans with a minimum increase in blood pressure of 10 mmHg measured in the arms. Additionally, using heart rate elevation as a marker of physiologic response to pain, our secondary study outcome is defined as the successful application of CAT tourniquets in normal physiology in healthy humans with no significant change in heart rate. By achieving both our primary and secondary outcomes, we can confidently attribute the elevation in blood pressure to the effects of the tourniquet application as opposed to a physiologic response to pain.

### Participant timeline

**Fig 1:**
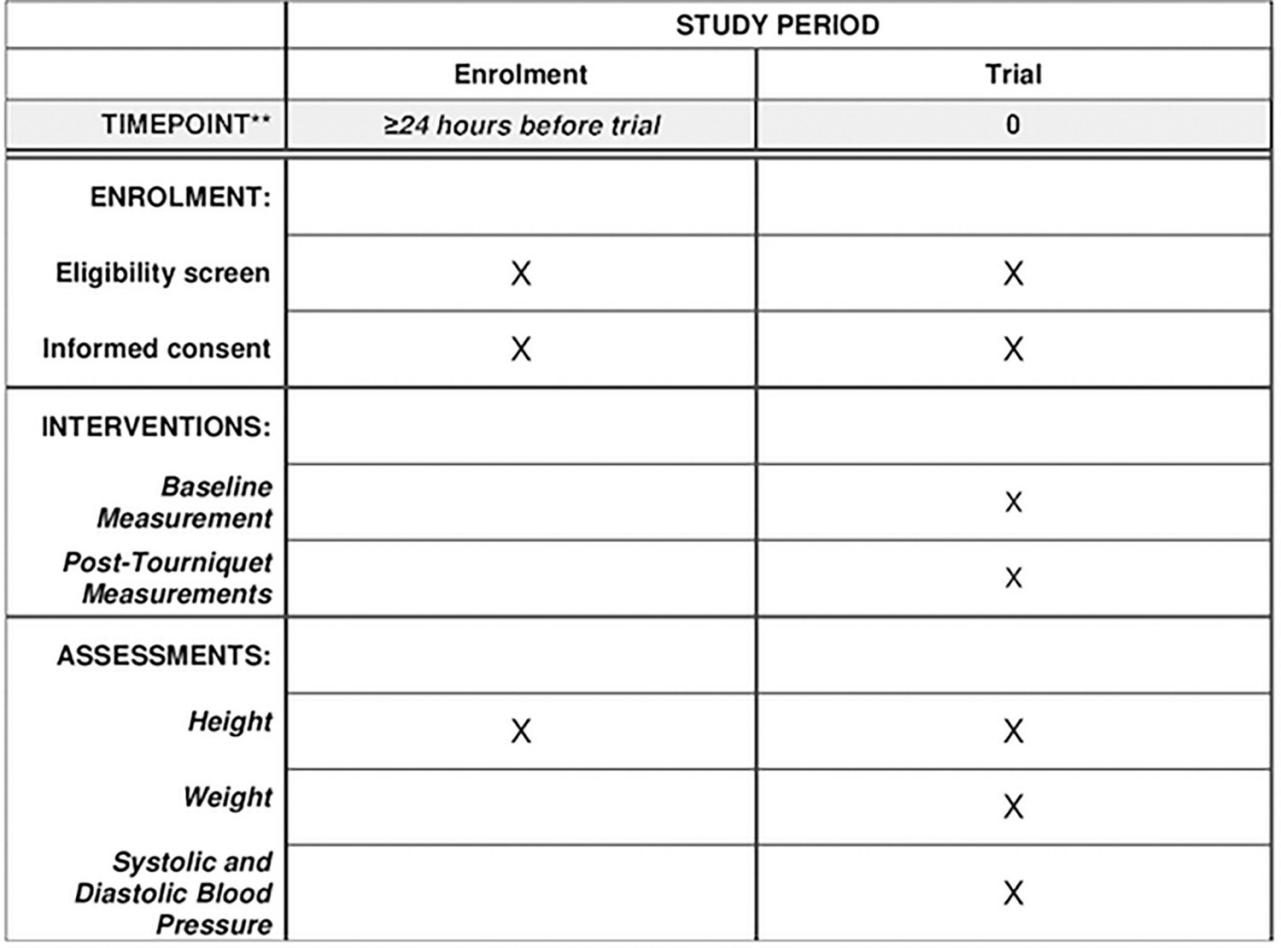
Schedule of enrollment, interventions, and assessments.

Volunteers identified as potential study participants will be informed of the study protocol and guidelines by a phone call at least 24 hours prior to the start of the study. Immediately following the phone call, volunteers will be emailed a copy of the consent form to review. On the day of the study, participants will be seen in the order that they arrive, and they will be consented in a private room by IRB-approved personnel. After being consented, they will have their baseline measurements taken. Then, they will undergo the three tourniquet trials with appropriate breaks between each trial. The informed consent, baseline measurements, and tourniquet trials will be done on the same day. No follow-up is needed (Fig 1).

### Sample size

A total of 30 healthy volunteered adults will be used for our study. A power analysis was conducted to determine appropriate sample size based on previous data examining hemodynamics following tourniquet application on the lower extremities [24]. Based on a power of 0.8 and an alpha of 0.05, a sample size of 24 participants will be needed for our study. Allowing for post hoc analysis and individual data exclusion if warranted, a total of 30 volunteer research participants will be recruited to partake in the study.

### Recruitment

Flyers will be displayed around the University campus in order to recruit subjects voluntarily. Volunteers will be screened to determine their ASA Class and will be asked questions regarding known chronic diseases and the exclusion criteria to identify potential subjects.

### Data collection: Trial procedures and evaluations

Data will be collected by IRB-approved personnel only. Blood pressure and heart rate will be measured using the OMRON 3 Series upper arm home blood pressure monitor BP7100. IRB-approved personnel will be trained on how to deploy the CAT tourniquets properly and safely on the lower extremities (i.e., thigh) and the automated sphygmomanometers on the upper extremities prior to the start of the study. Prior to deployment of either the CAT tourniquet or the automated sphygmomanometer, IRB-approved personnel will inspect the devices for any defects and sterilize the devices with 70% isopropyl alcohol or 70% ethanol. Following removal of the devices from the study participants, they will be sterilized with 70% isopropyl alcohol or 70% ethanol.

### Data management

All data associated with the research participants will be kept in a secure database with a password-protected server. The data will be coded, and a linking document containing any identifiers will be kept separate from the research data. Hard copies of the consent forms and any recorded data will be kept in a locked file cabinet with the key stored separately. Data may be used in future studies following IRB approval without additional informed consent from research participants.

### Statistical methods

Changes in systolic blood pressure, diastolic blood pressure, and heart rate will be analyzed using a Shapiro-Wilk test. Then, a one-way repeated measures analysis of variance (ANOVA) will be performed as well as a Holm-Sidak post-hoc test to determine the mean differences. The significance level will be set to 5% for statistical significance.

### Safety/Harms

The most common complications following application of a CAT are nerve injury, as a result of compression, and pain at the site of tourniquet application. Nerve injury usually occurs at the edges of the tourniquet due to the increased pressure gradient. Symptoms are typically transient resulting in mild to moderate pain. To increase the safety of the study and reduce the likelihood of complications, the tourniquet will be tightened to the minimum pressure needed to occlude blood flow to the lower extremities and will be removed after 5 minutes, since injury usually occurs 30–60 minutes following tourniquet application [25]. If 25% of subjects report nerve injury or pain at the tourniquet application site, the study will be stopped.

### Auditing

Auditing of the trial conduct will be conducted by the Institutional Representatives. The investigators and trial sponsor will have no role in the auditing process.

### Research ethics approval

The Institutional Review Board-II–Medical University of South Carolina has reviewed and approved this protocol (S1 Appendix) as well as the informed consent documents (S2 Appendix). The trial sponsor has also reviewed and approved the protocol and informed consent documents. Written consent will be obtained for each participant.

### Protocol amendments

Modifications made to the trial methods, site selection, informed consent documents, recruitment methods, safety assessments, risk factors, or any other aspects of the protocol will be reviewed and approved by the Institutional Review Board-II–Medical University of South Carolina as well as the trial sponsor.

### Ancillary and post-trial care

If a research subject is injured as a result of participation in the study, they will be advised to immediately go to the emergency room of the nearest hospital. The participant should tell the physician on call that they were part of a research study and may call the study doctor for details. If the study sponsor does not pay for the treatment, the healthcare facility who administered treatment will bill the research participant’s insurance company. If the insurance company denies coverage or the participant is uninsured, the research participant will be responsible for payment for all services administered. The research participants will be notified of these guidelines during voluntary informed consent.

### Dissemination of trial results

Results or data collected in the study will not be disclosed to study participants. Results will only become available to non-IRB-approved personnel after they are published in a peer-reviewed journal.

### Authorship

Eligibility for authorship requires IRB approval, participation in data collection, participation in data analysis, and participation in writing and/or reviewing the manuscript. There will be no professional writers employed.

### Status and timeline of study

This study is currently recruiting participants.

## Supporting information

S1 Checklist Complete spirit checklist. (DOC)

S1 Appendix IRB-approved protocol. (DOCX)

S2 Appendix Informed consent form. (DOCX)

## References

[1] Daley J; Morrison JJ; Sather J; Hile L. The role of resuscitative endovascular balloon occlusion of the aorta (REBOA) as an adjunct to ACLS in non-traumatic cardiac arrest. *Am J Emerg Med*. 2017;35(5):731–736. doi: 10.1016/j.ajem.2017.01.010 28117180

[2] Brenner M, Inaba K, Aiolfi A, DuBose J, Fabian T, Bee T, et al. Resuscitative Endovascular Balloon Occlusion of the Aorta and Resuscitative Thoracotomy in Select Patients with Hemorrhagic Shock: Early Results from the American Association for the Surgery of Trauma’s Aortic Occlusion in Resuscitation for Trauma and Acu. *J Am Coll Surg*. 2018;226(5):730–740. doi: 10.1016/j.jamcollsurg.2018.01.044 29421694

[3] Kong L, Yu Y, Li F, Cui H. Intra-Aortic Balloon Occlusion Decreases Blood Loss During Open Reduction and Internal Fixation for Delayed Acetabular Fractures: A Retrospective Study of 43 Patients. *J Investig Surg*. 2020;33(5):468–473. doi: 10.1080/08941939.2018.1516837 30395741

[4] Sesma J, Sara MJ, Espila JL, Arteche A, Saez MJ, Labandeira J. Effect of intra-aortic occlusion balloon in external thoracic compressions during CPR in pigs. *Am J Emerg Med*. 2002;20(5):453–462. doi: 10.1053/ajem.2002.32627 12216044

[5] Nozari A, Rubertsson S, Wiklund L. Improved cerebral blood supply and oxygenation by aortic balloon occlusion combined with intra-aortic vasopressin administration during experimental cardiopulmonary resuscitation. *Acta Anaesthesiol Scand*. 2000;44(10):1209–1219. doi: 10.1034/j.1399-6576.2000.441005.x 11065200

[6] Nozari A, Rubertsson S, Wiklund L. Intra-aortic administration of epinephrine above an aortic balloon occlusion during experimental CPR does not further improve cerebral blood flow and oxygenation. *Resuscitation*. 2000;44(2):119–127. doi: 10.1016/s0300-9572(00)00132-5 10767499

[7] Gedeborg R, Rubertsson S, Wiklund L. Improved haemodynamics and restoration of spontaneous circulation with constant aortic occlusion during experimental cardiopulmonary resuscitation. *Resuscitation*. 1999;40(3):171–180. doi: 10.1016/s0300-9572(99)00021-0 10395400

[8] Rubertsson S, Bircher NG, Alexander H. Effects of intra-aortic balloon occlusion on hemodynamics during, and survival after cardiopulmonary resuscitation in dogs. *Crit Care Med*. 1997;25(6):1003–1009. doi: 10.1097/00003246-199706000-00018 9201054

[9] Deakin CD. Intra-aortic administration of epinephrine above aortic occlusion does not alter outcome of experimental cardiopulmonary resuscitation. *Resuscitation*. 2000;44(1):75. doi: 10.1016/s0300-9572(99)00152-5 10777393

[10] Barton C, Manning JE, Batson N. Effect of selective aortic arch perfusion on median frequency and peak amplitude of ventricular fibrillation in a canine model. *Ann Emerg Med*. 1996;27(5):610–616. doi: 10.1016/s0196-0644(96)70165-8 8629783

[11] Paradis NA, Rose MI, Gawryl MS. Selective aortic perfusion and oxygenation: An effective adjunct to external chest compression-based cardiopulmonary resuscitation. *J Am Coll Cardiol*. 1994;23(2):497–504. doi: 10.1016/0735-1097(94)90439-1 8294706

[12] Rall JM, Ross JD, Clemens MS, Cox JM, Buckley TA, Morrison JJ. Hemodynamic effects of the Abdominal Aortic and Junctional Tourniquet in a hemorrhagic swine model. *J Surg Res*. 2017;212:159–166. doi: 10.1016/j.jss.2017.01.020 28550903

[13] Rall J, Cox JM, Maddry J. The use of the abdominal aortic and junctional tourniquet during cardiopulmonary resuscitation following traumatic cardiac arrest in swine. *Mil Med*. 2017;182(9):e2001– e2005. doi: 10.7205/MILMED-D-16-00409 28885969

[14] Hewitt CW, Pombo MA, Blough PE, Castaneda MG, Percival TJ, Rall JM. Effect of the Abdominal Aortic and Junctional Tourniquet on chest compressions in a swine model of ventricular fibrillation. *Am J Emerg Med*. 2020;Epub ahead. doi: 10.1016/j.ajem.2020.08.075 33046311

[15] Aslanger E, Goluck E, Oflaz H, Yilmaz A, Mercanoglu F, Bugra Z, et al. Intraaortic balloon occlusion during refractory cardiac arrest. A care report. *Resuscitation*. 2009;80(2):281–283. doi: 10.1016/j.resuscitation.2008.10.017 19058900

[16] Dogan EM, Beskow L, Calais F, Hörer TM, Axelsson B, Nilsson KF. Resuscitative Endovascular Balloon Occlusion of the Aorta in Experimental Cardiopulmonary Resuscitation: Aortic Occlusion Level Matters. *Shock*. 2019;52(1):67–74. doi: 10.1097/SHK.0000000000001236 30067564 PMC6587222

[17] Yang Z, Tang D, Wu X, Hu X, Xu J, Quian J, et al. A tourniquet assisted cardiopulmonary resuscitation augments myocardial perfusion in a porcine model of cardiac arrest. *Resuscitation*. 2015;86(49–53):49–53. doi: 10.1016/j.resuscitation.2014.10.009 25447436

[18] Brännström A, Rocksén D, Hartman J, Nyman N, Gustavsson J, Arborelius UP, et al. Abdominal aortic and junctional tourniquet release after 240 minutes is survivable and associated with small intestine and liver ischemia after porcine class II hemorrhage. *J Trauma Acute Care Surg*. 2018;85(4):717–724. doi: 10.1097/TA.0000000000002013 29985233

[19] Kheirabadi BS, Terrazas IB, Miranda N, Voelker AN, Klemcke HG, Brown AW, et al. Long-term consequences of abdominal aortic and junctional tourniquet for hemorrhage control. *J Surg Res*. 2018;231:99–108. doi: 10.1016/j.jss.2018.05.017 30278975

[20] Morgan R. W., French B., Kilbaugh T. J., Naim M. Y., Wolfe H., Bratinov G., et al. (2016). A quantitative comparison of physiologic indicators of cardiopulmonary resuscitation quality: Diastolic blood pressure versus end-tidal carbon dioxide. Resuscitation, 104, 6–11. doi: 10.1016/j.resuscitation.2016.04.004 27107688 PMC4902744

[21] Harper N. J. N., Nolan J. P., Soar J., Cook T. M. (2019). Why chest compressions should start when systolic arterial blood pressure is below 50 mm hg in the anaesthetised patient. British Journal of Anaesthesia, 124(3), 234–238. doi: 10.1016/j.bja.2019.11.005 31836135

[22] Bennett, M.E.a.J., *Tourniquet and Method of Use*. 2007: United States.

[23] Esposito, M., *Tourniquet and Method of Use*. 2005: United States.

[24] Bradford EM. Haemodynamic changes associated with the application of lower limb tourniquets. Anaesthesia. 1969 Apr;24(2):190–197. doi: 10.1111/j.1365-2044.1969.tb02837.x . 5774710

[25] Masri BA, Eisen A, Duncan CP, McEwen JA. Tourniquet-induced nerve compression injuries are caused by high pressure levels and gradients—a review of the evidence to guide safe surgical, pre-hospital and blood flow restriction usage. *BMC Biomed Eng*. 2020. 2:7. doi: 10.1186/s42490-020-00041-5 32903342 PMC7422508

