# Supplementary material for: A single-center, nonblinded, clinical trial comparing blood pressures before and after tourniquet application in healthy humans: A study protocol
Source: PLoS One. 2023 Jan 6;18(1):e0280139. doi: 10.1371/journal.pone.0280139 (PMC9821481; doi:10.1371/journal.pone.0280139)
Supplement: S1 Appendix — (DOCX) [file pone.0280139.s002.docx]

**PROTOCOL TITLE:**

Extremity Occlusion with Tourniquets Increases Proximal Systolic Pressure

**PRINCIPAL INVESTIGATOR:**

Dr. Taufiek Konrad Rajab

# Objectives / Specific Aims

We intend to demonstrate that non-invasive, peripheral vascular occlusion increases blood pressure in the conserved vasculature in healthy humans. Data from this study will provide proof of concept for the development of a novel device to improve cardiopulmonary resuscitation (CPR) outcomes during non-traumatic cardiac arrest. We hypothesize that peripheral vascular occlusion of non-vital vascular beds (the lower extremities) will increase blood flow to the more vital organs, the heart and the brain, leading to an improvement in survival and neurologic recovery after cardiac arrest as these outcomes are dependent on heart and brain ischemic injury. By using commercially available, FDA-approved, tourniquets on healthy humans to measure blood pressure with peripheral vascular occlusion, we will be testing our hypothesis in the safest way. We also will demonstrate no substantial change in heart rate.

# 2.0 Background

# For decades, the fields of trauma, orthopedics, and cardiology have utilized vascular occlusion devices for either hemorrhage control or to increase central pressure.^1-3^ Due to their success in maintaining blood flow proximally during shock scenarios, vascular occlusion devices have increasingly been studied during non-traumatic cardiac arrests. There have been nine preclinical studies in animals that have demonstrated endovascular balloon occlusion devices significantly increase coronary artery blood flow and pressure during cardiac arrest.^4-11^ Tourniquets are an external, non-invasive strategy to obtain the same end goal of vascular occlusion and are currently used daily in clinical practice for traumatic wounds and extremity surgery. They have been well studied in civilian and military research for their safety and efficacy, but their use as a CPR adjunct has yet to be realized.^12-14^

**Rationale:** To our knowledge, blood flow or pressure to vascular beds supplying vital organs (i.e., brain, heart) has not been measured in healthy adult subjects following administration of a non-invasive peripheral vascular occlusion device. This proof-of-concept data is necessary for future development and hypothesis testing for novel CPR adjuncts, which could improve survival and neurologic recovery following cardiac arrest.

1. Intervention to be studied

The fluid dynamics Hagen-Poiseuille Equation demonstrates in physical law why peripheral vasoconstriction increases the flow in conserved vascular networks. The Hagen-Poiseuille Equation (𝑸 = ^∆Pπ𝑟4^) gives the volumetric flow rate (𝑄) in a cylindrical tube as it relates to the viscosity of the fluid (𝜈), length of the system (𝐿), radius of the tube (𝑟), and pressure gradient across the tubing (∆𝑃). Tourniquets placed on the legs would decrease the length of the system (𝐿), thereby increasing the flow (𝑄) as these variables are inversely proportional. While this equation is an obvious oversimplification, we find it models our expectant results and confirms what we intuitively and anecdotally have noted in our patients. Beyond theory, studies in large animal models and clinical trials offer additional proof of concept.^4,12-13,15-19.^ Pigs receiving tourniquet-assisted CPR experienced significantly increased coronary blood flow, carotid artery pressure, systolic and diastolic blood pressure, end-tidal carbon dioxide, and survival than controls.^17^

In order to complete our hypothesis testing safely, we plan to deploy well-studied and FDA approved combat application tourniquets (CAT) on both thighs of volunteer healthy adult subjects while measuring blood pressure in the upper extremities. Research participants in this study will have their blood pressure measured in their upper extremities before and after tourniquet administration in order to serve as within subject controls. The tourniquets will be deployed for no longer than 5 minutes for each of the three repetitions. After the removal of the tourniquets, the participants will be allowed to rest for 3-5 minutes before the next application of the tourniquet.

CAT tourniquets were developed in 2005 by Mark Esposito et al. (US Patent numbers 7,842,067 and 7,892,253) and soon after obtained FDA approval. CAT tourniquets have been indicated for use in emergency situations revolving around a wounded limb, such as hemorrhage control.^22-23^ CAT tourniquets are the official tourniquet of the US Army since in 2005.

# 4.0 Study Endpoints (if applicable)

- - **Primary Study Endpoint:** A cardiac arrest porcine model that utilized an intra-aortic balloon catheter for occlusion of the aorta at the level of the diaphragm demonstrated a mean increase in systolic pressure of 17mmHg.^18^ Extrapolating from this data to our more distal point of occlusion at the lower extremities, we define the primary study endpoint as successful application of CAT tourniquets in normal physiology in healthy humans with a minimum increase in blood pressure of 10 mmHg measured in the arms. Another primary endpoint is demonstrating no significant change in participant heart rate.
  - **Primary Safety Endpoint:** The most common complications following application of a CAT tourniquet is pain or discomfort at the site of tourniquet application. This usually occurs at the edges of the tourniquet due to the increased pressure gradient. Symptoms are typically transient resulting in mild to moderate pain. In order to increase safety of the study and reduce the likelihood of complications, the tourniquet will be tightened to the minimum pressure needed to occlude blood flow to the lower extremities and will be removed after 5 minutes since injury usually occurs 30-60 minutes following tourniquet application^20^. If any of the subjects report intolerable pain during the study, their participation will be ended immediately.
  - **Secondary Safety Endpoints:** There are no secondary safety endpoints.

# 5.0 Inclusion and Exclusion Criteria/ Study Population

**Inclusion Criteria**

- - - - Healthy adults with no known chronic medical conditions between the ages of 18 and 60 years old willing and able to consent themselves.
      - Subjects who are native English speakers or self-report as fluent in English.

**Exclusion Criteria**

- - - - Subjects who have a history of or are a current smoker, have a history of or current illicit drug use, have a history of or current alcohol abuse.
      - Subjects who self-report as pregnant.
      - Subjects who self-report diabetes, cardiac disease including hypertension requiring medication, or a history of compartment syndrome or peripheral vascular disease.
      - Subjects with known chronic diseases not listed above, including but not limited to Rheumatoid Arthritis, McArdle’s disease, etc.
      - Subjects with elevated blood pressure (>130/80mmHg) on the day of the study

# 6.0 Number of Subjects

- A total of 30 healthy volunteered adults (15 males and 15 females) will be used for our study.
- A power analysis was conducted to determine appropriate sample sizes based on previous data examining hemodynamics following tourniquet application on the lower extremities^21^. Based on a power of 0.8 and an alpha of 0.05, a sample size of 24 participants will be needed for our study. Allowing for post hoc analysis and individual data exclusion if warranted, a total of 30 volunteer research participants will be recruited to partake in the study.

# 7.0 Setting

- - Study participants will meet in a private conference room at MUSC where the research protocol will be completed.

**Study Sites**

- - MUSC

# 8.0 Recruitment Methods

- - Flyers will be displayed around the MUSC campus in order to recruit subjects voluntarily (Copy of flyer attached to IRB application).
  - Volunteers will be screened via a phone call to determine their eligibility for the study and will be asked questions related to the exclusion criteria in order to identify potential subjects.

# 9.0 Consent Process

- - - - IRB approved personnel will obtain voluntary informed consent for all research participants.
      - Volunteers that were identified as potential subjects based on inclusion and exclusion criteria will be informed of the study procedures over the phone prior to participation in the study.
      - Participants will be consented the day of the study in a private room by IRB approved personnel prior to beginning the study. This will be conducted in person through a signed written consent form. Participants will have ample time to read the consent form and ask questions to IRB approved personnel.
      - Participants may refuse to take part in the study or stop taking part in the study at any time by notifying the study investigator or approved IRB personnel.
      - There will be no cost nor compensation for participating in the study to avoid undue influence or coercion of study participants.

# 10.0 Study Design / Methods

After expressing interest in the study, participants will be asked to come to MUSC for the study visit. On the day of the study, each volunteer will be taken to a private room by IRB approved personnel and consented in person through a signed written consent form. They will be asked to confirm their eligibility in the study based on the inclusion/exclusion criteria. Their blood pressure in their upper extremity will be taken to ensure that it is not elevated on the day of the study (must not be >130/80mmHg). Their responses and blood pressure will be documented. Once the participants have consented to the study, they will have their height and weight recorded. Then, each participant will have their blood pressure measured in each arm by an automated sphygmomanometer while lying down on a medical bench. The blood pressures and the heart rate will be recorded by IRB approved personnel. This will allow each research participant to act as their own control for the experimental procedure. Then, one CAT tourniquet will be placed around each thigh and tightened until no pulse can be felt in the lower extremity. Blood pressure measurements will be repeated in each arm once the tourniquets are secured. Once these blood pressures are documented, the tourniquets will be removed. The maximum time the tourniquets will be applied is 5 minutes to ensure ample time for blood pressure recording. If the measurement cannot be taken within the time frame, the tourniquets will be released. The participants will rest 3-5 minutes between each repetition of the tourniquet application. The whole process will be repeated three times for data validity. CAT tourniquets have been extensively studied for safety, and no long-term consequences or complications have been noted for up to two hours of continuous use. This team will prioritize the safety and comfort of the participant and will limit the tourniquet application time to a maximum of 5 minutes. We will be using the FDA-approved Combat Application Tourniquet (CAT) tourniquet (North American Rescue, Greer, South Carolina, USA). After the data is collected, a debrief session will be conducted, allowing for additional subject monitoring after removal of the tourniquets. During the debrief session, the participant will be asked to lay down or sit in a relaxed position for 5 minutes or until they feel that their leg function is back to baseline. If there is any concern from the participant, the PI or IRB-approved personnel who are medical doctors, will conduct an evaluation. If any of the safety endpoints are noted, it will be recorded, and the participant will be taken to the emergency room for full examination and work-up. The PI or IRB-approved personnel will follow-up with the participant remotely until the safety concern is resolved.

# 11.0 Data Management

- - All data associated with the research participants will be kept in a secure database with a password protected server. The data will be coded and a linking document containing any identifiers will be kept separate from the research data.
  - Hard copies of the consent forms and any recorded data will be kept in a locked file cabinet with the key stored separately.
  - Data may be used in future studies following IRB approval without additional informed consent from research participants.

# 12.0 Withdrawal of Subjects

- - - Research subjects may refuse to participate or stop participating in the study at any time by notifying the principal investigator or approved IRB personnel.
    - If research participants would like to have their data removed from the study, they must notify the principal investigator in writing within a week of completing the study.
    - If the principal investigator or approved IRB personnel determine that participation in the study is not in the best interest of the research subject, they may remove the research participant from the study.

# 13.0 Risks to Subjects

- - - There is a possibility of pain or discomfort at the site of tourniquet application following deployment of the tourniquet. Symptoms are typically transient and result in mild to moderate pain, numbness, or tingling. Injury typically occurs at the edges of the tourniquet application due to the increased pressure gradient. Risk for injury increases with duration of tourniquet application (>60 minutes) and the tightness of the tourniquet applied. CAT tourniquets will be deployed for less than 5 minutes and will be tightened up to the necessary pressure to occlude distal blood flow.
    - If the above occurs during the research study, IRB approved personnel will discontinue testing for that individual and recommend that the research participant seek medical assistance at the closet emergency room. IRB-approved personnel or PI will follow up with that individual remotely until the injury is resolved.
    - There is a risk for injury/discomfort to the skin or subcutaneous tissue at the site of tourniquet or sphygmomanometer application such as, bruising or petechiae. These symptoms are transient and are typically mild.
    - There is a potential risk for loss of confidentiality in the unlikely event of a data leakage.

# 14.0 Potential Benefits to Subjects or Others

The research participants will receive a small monetary benefit of $10 for their participation.

# 15.0 Sharing of Results with Subjects

Results or data collected in the study will not be disclosed to study participants.

# 16.0 Drugs or Devices

CAT tourniquets and automated sphygmomanometers will be purchased by the Principal Investigator and stored in designated laboratory or office space of the principal investigator. IRB approved personnel will be trained on how to properly and safely deploy the CAT tourniquets on the lower extremities (i.e., thigh) and the automated sphygmomanometers on the upper extremities prior to the start of the study. Prior to deployment of either the CAT tourniquet or the automated sphygmomanometer, IRB approved personnel will inspect the devices for any defects and sterilize the devices with 70% isopropyl alcohol or 70% ethanol. Following removal of the devices from the study participants, they will be sterilized with 70% isopropyl alcohol or 70% ethanol.

# References

1. Daley, J; Morrison, JJ; Sather, J; Hile L. The role of resuscitative endovascular balloon occlusion of the aorta (REBOA) as an adjunct to ACLS in non-traumatic cardiac arrest. *Am J Emerg Med*. 2017;35(5):731-736. doi:10.1016/j.ajem.2017.01.010

2. Brenner M, Inaba K, Aiolfi A, et al. Resuscitative Endovascular Balloon Occlusion of the Aorta and Resuscitative Thoracotomy in Select Patients with Hemorrhagic Shock: Early Results from the American Association for the Surgery of Trauma’s Aortic Occlusion in Resuscitation for Trauma and Acu. *J Am Coll Surg*. 2018;226(5):730-740. doi:10.1016/j.jamcollsurg.2018.01.044

3. Kong L, Yu Y, Li F, Cui H. Intra-Aortic Balloon Occlusion Decreases Blood Loss During Open Reduction and Internal Fixation for Delayed Acetabular Fractures: A Retrospective Study of 43 Patients. *J Investig Surg*. 2020;33(5):468-473. doi:10.1080/08941939.2018.1516837

4. Sesma J, Sara MJ, Espila JL, Arteche A, Saez MJ, Labandeira J. Effect of intra-aortic occlusion balloon in external thoracic compressions during CPR in pigs. *Am J Emerg Med*. 2002;20(5):453-462. doi:10.1053/ajem.2002.32627

5. Nozari A, Rubertsson S, Wiklund L. Improved cerebral blood supply and oxygenation by aortic balloon occlusion combined with intra-aortic vasopressin administration during experimental cardiopulmonary resuscitation. *Acta Anaesthesiol Scand*. 2000;44(10):1209-1219. doi:10.1034/j.1399- 6576.2000.441005.x

6. Nozari A, Rubertsson S, Wiklund L. Intra-aortic administration of epinephrine above an aortic balloon occlusion during experimental CPR does not further improve cerebral blood flow and oxygenation. *Resuscitation*. 2000;44(2):119-127. doi:10.1016/S0300-9572(00)00132-5

7. Gedeborg R, Rubertsson S, Wiklund L. Improved haemodynamics and restoration of spontaneous circulation with constant aortic occlusion during experimental cardiopulmonary resuscitation. *Resuscitation*. 1999;40(3):171-180. doi:10.1016/S0300-9572(99)00021-0

8. Rubertsson S, Bircher NG, Alexander H. Effects of intra-aortic balloon occlusion on hemodynamics during, and survival after cardiopulmonary resuscitation in dogs. *Crit Care Med*. 1997;25(6):1003-1009. doi:10.1097/00003246-199706000-00018

9. Deakin CD. Intra-aortic administration of epinephrine above aortic occlusion does not alter outcome of experimental cardiopulmonary resuscitation. *Resuscitation*. 2000;44(1):75. doi:10.1016/S0300- 9572(99)00152-5

10. Barton C, Manning JE, Batson N. Effect of selective aortic arch perfusion on median frequency and peak amplitude of ventricular fibrillation in a canine model. *Ann Emerg Med*. 1996;27(5):610-616. doi:10.1016/S0196-0644(96)70165-8

11. Paradis NA, Rose MI, Gawryl MS. Selective aortic perfusion and oxygenation: An effective adjunct to external chest compression-based cardiopulmonary resuscitation. *J Am Coll Cardiol*. 1994;23(2):497- 504. doi:10.1016/0735-1097(94)90439-1

12. Rall JM, Ross JD, Clemens MS, Cox JM, Buckley TA, Morrison JJ. Hemodynamic effects of the Abdominal Aortic and Junctional Tourniquet in a hemorrhagic swine model. *J Surg Res*. 2017;212:159- 166. doi:10.1016/j.jss.2017.01.020

13. Rall J, Cox JM, Maddry J. The use of the abdominal aortic and junctional tourniquet during cardiopulmonary resuscitation following traumatic cardiac arrest in swine. *Mil Med*. 2017;182(9):e2001- e2005. doi:10.7205/MILMED-D-16-00409

14. Hewitt CW, Pombo MA, Blough PE, Castaneda MG, Percival TJ, Rall JM. Effect of the Abdominal Aortic and Junctional Tourniquet on chest compressions in a swine model of ventricular fibrillation. *Am J Emerg Med*. 2020;Epub ahead. doi:10.1016/j.ajem.2020.08.075

15. Aslanger, E; Goluck, E; Oflaz, H; Yilmaz, A; Mercanoglu, F; Bugra, Z; Umman, B; Nisanci Y. Intraaortic balloon occlusion during refractory cardiac arrest. A care report. *Resuscitation*. 2009;80(2):281-283. doi:10.1016/j.resuscitation.2008.10.017

16. Dogan EM, Beskow L, Calais F, Hörer TM, Axelsson B, Nilsson KF. Resuscitative Endovascular Balloon Occlusion of the Aorta in Experimental Cardiopulmonary Resuscitation: Aortic Occlusion Level Matters. *Shock*. 2019;52(1):67-74. doi:10.1097/SHK.0000000000001236

17. Yang Z, Tang D, Wu X, et al. A tourniquet assisted cardiopulmonary resuscitation augments myocardial perfusion in a porcine model of cardiac arrest. *Resuscitation*. 2015;86(49-53):49-53. doi:10.1016/j.resuscitation.2014.10.009

18. Brännström A, Rocksén D, Hartman J, et al. Abdominal aortic and junctional tourniquet release after 240 minutes is survivable and associated with small intestine and liver ischemia after porcine class II hemorrhage. *J Trauma Acute Care Surg*. 2018;85(4):717-724. doi:10.1097/TA.0000000000002013

19. Kheirabadi BS, Terrazas IB, Miranda N, et al. Long-term consequences of abdominal aortic and junctional tourniquet for hemorrhage control. *J Surg Res*. 2018;231:99-108. doi:10.1016/j.jss.2018.05.017

20. Masri, B.A., et al., *Tourniquet-induced nerve compression injuries are caused by high pressure levels and gradients - a review of the evidence to guide safe surgical, pre-hospital and blood flow restriction usage.* BMC Biomed Eng, 2020. **2**: p. 7.

21. Bradford EM. Haemodynamic changes associated with the application of lower limb tourniquets. Anaesthesia. 1969 Apr;24(2):190-7. doi: 10.1111/j.1365-2044.1969.tb02837.x. PMID: 5774710.

22. Bennett, M.E.a.J., *Tourniquet and Method of Use*. 2007: United States.

23. Esposito, M., *Tourniquet and Method of Use*. 2005: United States.
