# Supplementary material for: A single-center, nonblinded, clinical trial comparing blood pressures before and after tourniquet application in healthy humans: A study protocol
Source: PLoS One. 2023 Jan 6;18(1):e0280139. doi: 10.1371/journal.pone.0280139 (PMC9821481; doi:10.1371/journal.pone.0280139)
Supplement: S2 Appendix — (DOCX) [file pone.0280139.s003.docx]

**Medical University of South Carolina CONSENT TO BE A RESEARCH SUBJECT**

**TITLE OF RESEARCH:** Extremity Occlusion with Tourniquets Increases Proximal Systolic Pressure

You are being asked to volunteer for a research study. Research studies are voluntary and include only people who choose to take part. This is a research study to find out if stopping blood flow to the legs with two tourniquets increases the blood pressure where blood flow is not stopped. The purpose of this is to show that doing this can increase the blood pressure in the heart and brain during cardiopulmonary resuscitation (CPR) to improve survival and recovery since these outcomes depend on blood flow to these organs.

You will fill out your height, weight, sex, and age upon enrollment. You will then have your blood pressure and heart rate taken with a standard, automated blood pressure cuff in both arms while lying down. Next, two tourniquets will be placed around both of your thighs. Once the tourniquets are secured, your blood pressure and heart rate will be taken in each arm again. The tourniquets will be released once the vitals are recorded or after 5 minutes. We will perform three repetitions of this. You will have an opportunity to describe your experience to the researcher as a debrief.

There will be no direct benefit to you from participating in this study. However, it is hoped that the information gained from the study will help in the treatment of future patients’ heart attack. Your participation will help the researchers develop a CPR device to help improve survival and recovery.  There are risks to participation, which include discomfort in one or both legs when the tourniquets are being used, discomfort in one or both arms when measurments are taken, tingling and numbness in one or both legs when the tourniquets are being used.  You do not have to participate in this study, participation is completely voluntary.  At any point, you can withdraw from the study.

If you are interested in learning more about this study, please continue reading below.

**PURPOSE OF THE RESEARCH**

Our goal in this study is to see if tourniquets around the legs that stop blood flow to the legs increase the blood pressure in the blood vessels that still have blood flow going through them. If the blood pressure increases in these places, we expect that the heart and brain are also having higher blood pressures, which would be important during a heart attack. Increased blood flow to the heart and brain during a heart attack can help improve survival and brain function. This study involves research of this concept. You are being asked to join to provide evidence for this hypothesis. The tourniquets we will be using for this study are FDA-approved for use to stop blood flow in the legs. We plan to have a total of 30 participants in this study.

Please read this consent form carefully and take your time making your decision. As your study doctor or study staff discusses this consent form with you, please ask him/her to explain any words or information that you do not clearly understand.  You are being asked to participate in this study because you are a person in good health. The investigator in charge of this study at MUSC is Dr. T. Konrad Rajab at 843-870-1950. The study is being done at one site. Approximately 30 people will take part in this study.

**PROCEDURES**

First, we will collect information on your age and general health status, to ensure you meet eligibility criteria. We will have you lie down and take your blood pressure in each arm. Then, we will secure a tourniquet on each of your legs while you’re lying down and repeat the blood pressure measurements in each arm. Blood pressure will be taken within one minute after the tourniquets are secured. Once the blood pressure measurements are taken, the tourniquets will be released. You will fill out a short questionnaire about your experience while the tourniquets were in use.

If you agree to be in this study, the following will happen:

1. You will have your age, sex, height, and weight recorded confidentially. Only participants that are healthy and between the ages of 18 and 60 years old can participate. Participants cannot have significant health problems which include, but are not limited to heart disease, COPD, chronic kidney disease, liver disease, and peripheral artery disease.
2. If you are eligible, you will lie down and have your blood pressure and heart rate taken in each arm.
3. Then, a tourniquet will be secured to each of your legs while you’re lying down, and blood pressure and heart rate measurements will be repeated in each arm. Once the blood pressure and heart rate measurements are taken, the tourniquets will be released. This process will repeat three times for validation purposes.
4. You will debrief with the study team and can fill out the questionnaire about your experience while the tourniquets were in use.

**DURATION**

Participation in the study will only be this one visit for about 15-30 minutes.

**MEDICAL RECORDS and/or CERTIFICATE OF CONFIDENTIALITY**

Information about your study participation will not be in your medical record.  This means that neither your research participation nor any of your research results will be included in any MUSC medical record.

**COSTS**

There will be no cost to you as a result of participation in this study.

**PAYMENT TO PARTICIPANTS**

You will not be paid for participating in this study.

**ALTERNATIVES**

Your alternative is to not participate in this study.

**DATA SHARING**

Information about you (including your identifiable private information and/or any identifiable biospecimens) may have all of your identifiers removed and used for future research studies or distributed to other researchers for future research without additional informed consent from you or your legally authorized representative.

**DISCLOSURE OF RESULTS**

Research results will not be disclosed to subjects.

**STUDENT PARTICIPATION**

Your participation or discontinuance will not constitute an element of your academic performance, nor will it be a part of your academic record at this Institution.

**EMPLOYEE PARTICIPATION**

Your participation or discontinuance will not constitute an element of your job performance or evaluation, nor will it be a part of your personnel record at this Institution.

**FUTURE CONTACT**

The researcher in charge of this study might like to contact you in the future about other research opportunities. Please initial by your choice below:

____Yes, I agree to be contacted

____No, I do not agree to be contacted

Results of this research will be used for the purposes described in this study. This information may be published, but you will not be identified. Information that is obtained concerning this research that can be identified with you will remain confidential to the extent possible within State and Federal law. The investigators associated with this study, the sponsor, and the MUSC Institutional Review Board for Human Research will have access to identifying information. All records in South Carolina are subject to subpoena by a court of law.

In the event that you are injured as a result of participation in this study, you should immediately go to the emergency room of the Medical University Hospital, or in case of an emergency go to the nearest hospital, and tell the physician on call that you are in a research study. They will call your study doctor who will make arrangements for your treatment. If the study sponsor does not pay for your treatment, the Medical University Hospital and the physicians who render treatment to you will bill your insurance company. If your insurance company denies coverage or insurance is not available, you will be responsible for payment for all services rendered to you.

Your participation in this study is voluntary. You may refuse to take part in or stop taking part in this study at any time. You should call the investigator in charge of this study if you decide to do this. Your decision not to take part in the study will not affect your current or future medical care or any benefits to which you are entitled.

The investigators and/or the sponsor may stop your participation in this study at any time if they decide it is in your best interest. They may also do this if you do not follow the investigator’s instructions.

**Volunteers Statement**

I have been given a chance to ask questions about this research study. These questions have been answered to my satisfaction. If I have any more questions about my participation in this study or study related injury, I may contact Dr. T. Konrad Rajab at 617-901-2638. I may contact the Medical University of SC Patient and Family Care Liaison (843) 792-5555 concerning medical treatment.

If I have any questions, problems, or concerns, desire further information or wish to offer input, I may contact the Medical University of SC Institutional Review Board for Human Research IRB Manager or the Office of Research Integrity Director at (843) 792-4148. This includes any questions about my rights as a research subject in this study.

I agree to participate in this study. I have been given a copy of this form for my own records.

________________________________

Signature of Person Obtaining Consent Date *Name of Participant

Signature of Participant Date

Participant’s Personal Representative (if applicable):

___________________________________________________

Name of Personal Representative *(Please print)*

___________________________________________________

Signature of Personal Representative Date
